# Establishment of Yan-Zhang's staging of digestive tract magnetic compression anastomosis in a rat model

**DOI:** 10.1038/s41598-022-16794-z

**Published:** 2022-07-20

**Authors:** Miaomiao Zhang, Xin Lyu, Guangbin Zhao, Yingfeng An, Yi Lyu, Xiaopeng Yan

**Affiliations:** 1 Department of Hepatobiliary Surgery, The First Affiliated Hospital of Xi’an Jiaotong University, 277 West Yanta Road, Xi’an, 710061 Shaanxi China; 2 National and Local Joint Engineering Research Center of Precision Surgery & Regenerative Medicine, The First Affiliated Hospital of Xi’an Jiaotong University, 277 West Yanta Road, Xi’an, 710061 Shaanxi China; 3 Department of Pulmonary and Critical Care Medicine, Second Affiliated Hospital, Xi’an Jiaotong University, Xi’an, 710004 Shaanxi China; 4 State Key Laboratory for Manufacturing System Engineering, School of Mechanical Engineering, Xi’an Jiaotong University, Xi’an, 710054 Shaanxi China; 5 Drug Non-Clinical Evaluation Center of Guangzhou Institute of Pharmaceutical Industry, Guangzhou General Pharmaceutical Research Institute Co. Ltd., Guangzhou, Guangdong China

**Keywords:** Colorectal surgery, Reconstruction, Gastrointestinal system

## Abstract

Magnetic compression anastomosis, also known as magnamosis, is a safe and feasible method for digestive tract anastomosis. However, the pathological process involved in magnamosis of the digestive tract has not been investigated. This study aimed to establish the stages of digestive tract magnamosis in a rat model. Eighty-four Sprague–Dawley albino rats (200–250 g) were randomly divided into 14 groups (n = 6 per group). All rats underwent colonic magnamosis. Starting from postoperative day (POD) 1, one group of rats was sacrificed every other day to obtain the specimens. Burst pressure at the anastomotic site of each specimen was examined. Gross and histological examination of the anastomotic site was performed to establish the stages of the digestive tract magnamosis. Colonic magnamosis was successfully performed in all rats and the mean anastomosis time was 5.62 ± 0.91 min. The postoperative survival rate was 100%. The lowest anastomotic burst pressure was 78.33 ± 3.44 mmHg on POD3. The anastomotic burst pressure gradually increased and stabilized on POD21. Macroscopic and histological examination showed that the anastomotic mucosal and serosal layer did not heal on POD1. The serosal layer of the anastomosis healed by adhesion on POD3, and the mucosal layer began to heal on POD3-11 and was established by POD21. According to the anastomotic bursting pressure, digestive tract magnamosis can be staged into the magnetic maintenance, fragile, strengthening, and stable phases, which on histology correspond to the serosal adhesion formation, serosal healing, mucosal healing, and stereotyping, respectively.

## Introduction

Magnetic compression anastomosis (MCA) is an emerging surgical technique that uses a specially designed magnetic device and non-contact magnetic field force between magnets to reconstruct the adjoining hollow viscus. MCA is currently being used for esophago-gastric anastomosis^1^, gastro-intestinal anastomosis^2,3^, small intestine anastomosis^4,5^, colonic anastomosis^6,7^, and rectovaginal fistula closure repair^8^. MCA has a wide range of applications and can be used during open, laparoscopic, robotic, and endoscopic operations^9–11^. It is the third commonly used technique of anastomosis after hand-sewn and stapled anastomosis. Compared to hand-sewn and stapled anastomosis, MCA is simpler and more reliable, with wider applications. Although MCA has been used in clinical practice for more than 30 years, there is a lack of in-depth and systematic studies on the histopathological changes in the tissue and the process of healing with MCA.

During MCA of the digestive tract, the daughter and parent magnets are usually located in the lumen. Under a continuous magnetic force field, the compressed intestinal wall between the magnets is believed to undergo a pathological process of ischemia-necrosis-shedding, while the intestinal wall tissue adjacent to the magnets is thought to undergo adherence-repair-healing to form the anastomosis^12^. However, the changes in the anastomotic tissue tension before and after the magnet falls off and the histopathological changes in the digestive tract during MCA are poorly understood.

In this study, we established a rat model to understand the dynamic changes in burst pressure of the anastomotic stoma during colonic MCA and investigated the corresponding histological changes in the mucosal and serous layers.

## Materials and methods

### Ethical statement

The ethics committee of Xi’an Jiaotong University approved this study (permit number: 2018-001). The research protocol and all the experimental procedures were strictly in accordance with the Guidelines for the Care and Use of Experimental Animals issued by the Xi’an Jiaotong University Medical Center.

### Study design

Eighty-four Sprague–Dawley albino rats (weight, 200–250 g) were bought from the Experimental Animal Center, College of Medicine, Xi’an Jiaotong University (Xi’an, China). The animals were acclimatized to laboratory conditions (23 °C, 12 h/12 h light/dark, 50% humidity, ad libitum access to food and water) for one week prior to commencing the experiments. The animal protocol was designed to minimize discomfort to the animals. The rats were randomly divided into 14 groups (n = 6 per group). Groups were named according to the postoperative day (POD) on which they were sacrificed (D1, D3, D5, and so on up to D27). All rats underwent colonic MCA. As this was a pilot study, all animals were included in the experimental group, and there was no control group. Intramuscular injection of pethidine hydrochloride (1 mg/kg) was administered every 12 h for analgesia for three days after surgery. At the end of the study, all animals were euthanized by a barbiturate overdose (intraperitoneal injection, 60 mg/kg pentobarbital sodium) for tissue collection.

### Magnetic anastomosis device

**Figure 1:**
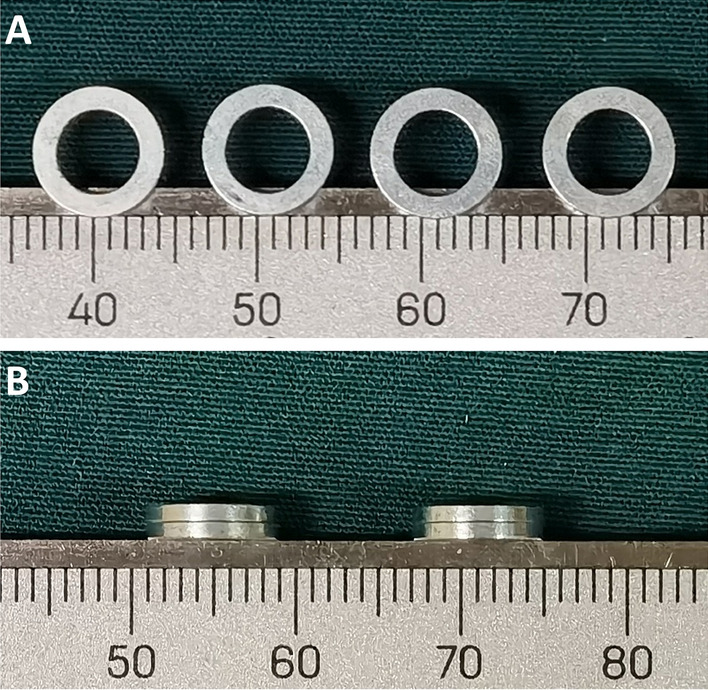
Magnetic rings. (**A**) Frontal view of the magnetic rings. (**B**) Side view of the magnetic rings attracted together.

The magnetic anastomosis rings were designed according to the anatomical characteristics of the rat colon. The thickness, external diameter, and internal diameter of the magnetic anastomosis rings were 1 mm, 8 mm, and 5 mm, respectively (Fig. 1). The rings were made of neodymium-iron-boron (grade N45) and were plated with zinc to increase resistance to erosion. Each ring weighed 0.2 g and had a magnetic density of 0.26 T.

### Surgical procedures

**Figure 2:**
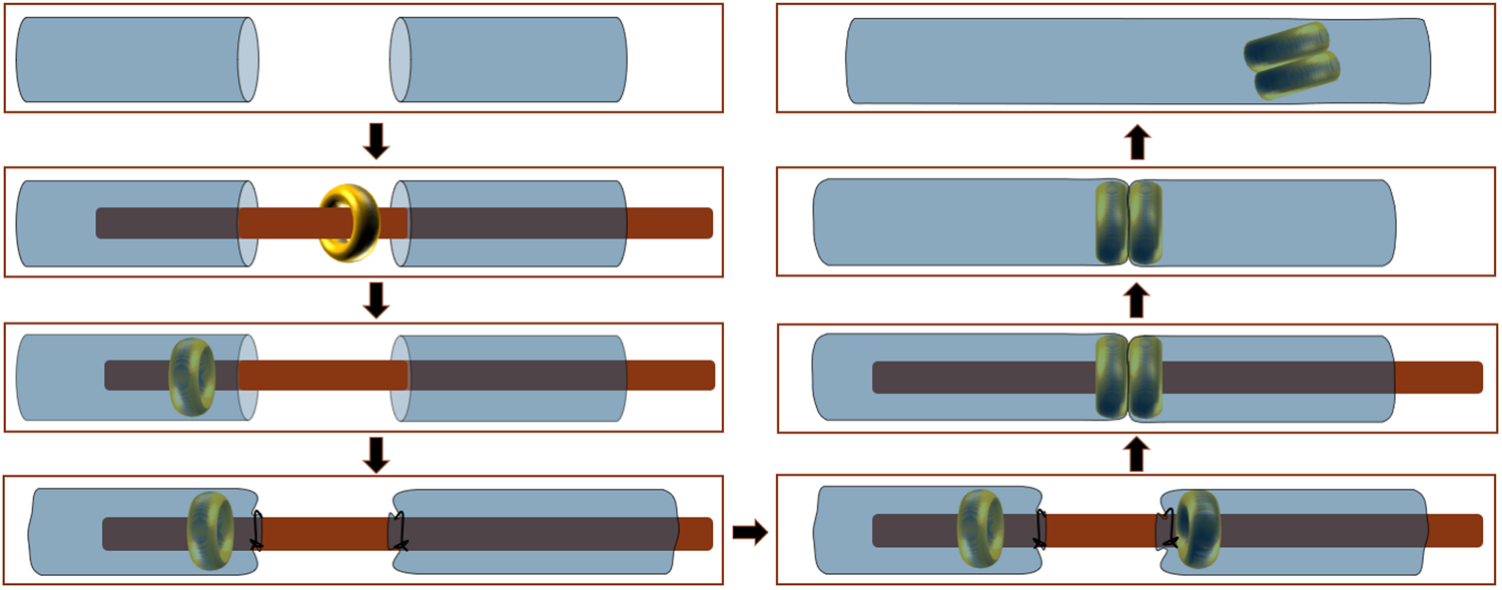
Schematic presentation of the colonic anastomosis using magnetic compression^13^.

After being fasted for 1 day, the rats were weighed and then anesthetized using an intraperitoneal injection of 3% pentobarbital sodium solution (0.1 mL/100 g). After confirming that the paw withdrawal reflex was absent, the animal was fixed in the supine position. Sterile surgical instruments were used throughout the procedure. The abdominal region of the rat was shaved, and a 3-cm-long midline abdominal incision was made. The end-to-end descending colonic (about 5–6 cm from the anus) anastomosis was performed using the magnetic anastomosis rings. The operation was carried out as described in our previous study (Fig. 2)^13^.

### Postoperative care

All rats were maintained in a single cage after surgery. After recovery from anesthesia, they were allowed only liquids and no standard chow. During the first three PODs, the rats were fed a liquid diet, and thereafter received normal chow. The rats’ general condition, eating habits, and magnet discharge time were observed daily.

The rats were sacrificed on the PODs corresponding to their group name to obtain the anastomotic specimens. For example, rats in the D1 group were sacrificed on POD1, those in the D3 group were sacrificed on POD3, and so on. The specimens were tested for burst pressure. The gross and histological changes at the anastomotic site were also studied.

### Measurement of burst pressure

The rats were euthanized using high-dose barbiturates. The burst pressure of the colonic anastomosis was measured using a pressure gauge. An 8-cm colonic segment containing the anastomotic site was resected. The proximal end of the colon was clamped using hemostatic forceps. A catheter was introduced through the anus, and a single silk suture was used to securely ligate the anus around the catheter. The entire colon specimen was completely immersed in 0.9% saline. The colonic pressure was gradually increased by pumping air into the catheter. The pressure at which the first air bubble escaped from the anastomotic site was recorded as the burst pressure.

### Specimen collection and histological analysis

All anastomosis-bearing colonic segments having sufficient length on either side of the anastomosis were harvested. After gross examination, all samples were immersed in 10% buffered formalin overnight. The fixed colonic segment was paraffin embedded, sliced into 4-μm-thin sections at the anastomosis site, stained with hematoxylin and eosin or Masson trichrome stain, and examined using bright-field microscopy.

### Statistical analysis

Data were analyzed using the SPSS statistical software package (*v*20.0). All quantitative data are presented as mean and standard deviation. The independent-sample *t*-test was used to compare the groups. Differences were considered to be statistically significant at *P* value of < 0.05.

## Results

### Survival rate and postoperative complications

The colonic MCA operation was successfully performed in all 84 rats. The mean anastomosis time was 5.62 ± 0.91 min and the survival rate was 100%. No cases of anastomotic leakage or stenosis were observed. The magnetic rings were expelled from the anus in 69 rats after 5.97 ± 1.12 days (range, 4–8 days). During the process of obtaining the anastomotic specimen, we found that a portion of the magnets were not expelled from the anus as follows. In the D1 group, the magnetic rings of all 6 rats were firmly located at the anastomosis when the specimen was obtained. In the D3 group, the magnetic rings of 3 rats were located at the anastomosis, but the rings fell off after a slight external force was applied. The magnetic rings in the remaining 3 rats in the D3 group were located in the rectum, but not outside of the anus. In the D5 and D7 groups, the magnets fell off into the rectum in 2 and 1 rat, respectively.

### Burst pressure

**Figure 3:**
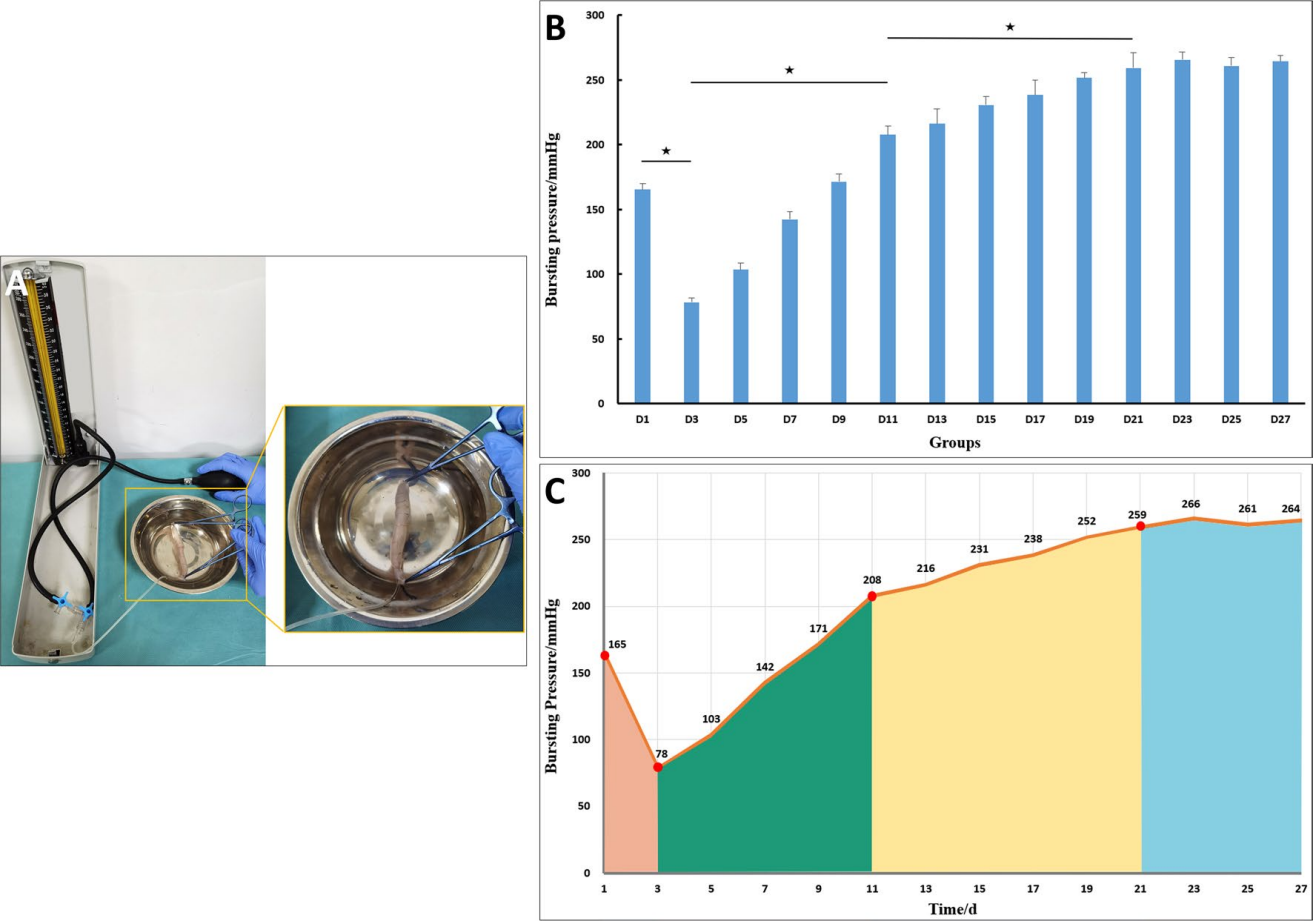
Burst pressure. (**A**) Schematic diagram of burst pressure measurement. (**B**) Bar graph of the mean burst pressure for each group. (“★” shows *P* < 0.001) (**C**) Line graph of the mean burst pressure for each group.

Burst pressure for each group of rats is shown in Fig. 3. The lowest burst pressure was in the D3 group (78.33 ± 3.44 mmHg). With time, the burst pressure of the anastomosis gradually increased and stabilized at POD21.

### Gross and microscopic findings

**Figure 4:**
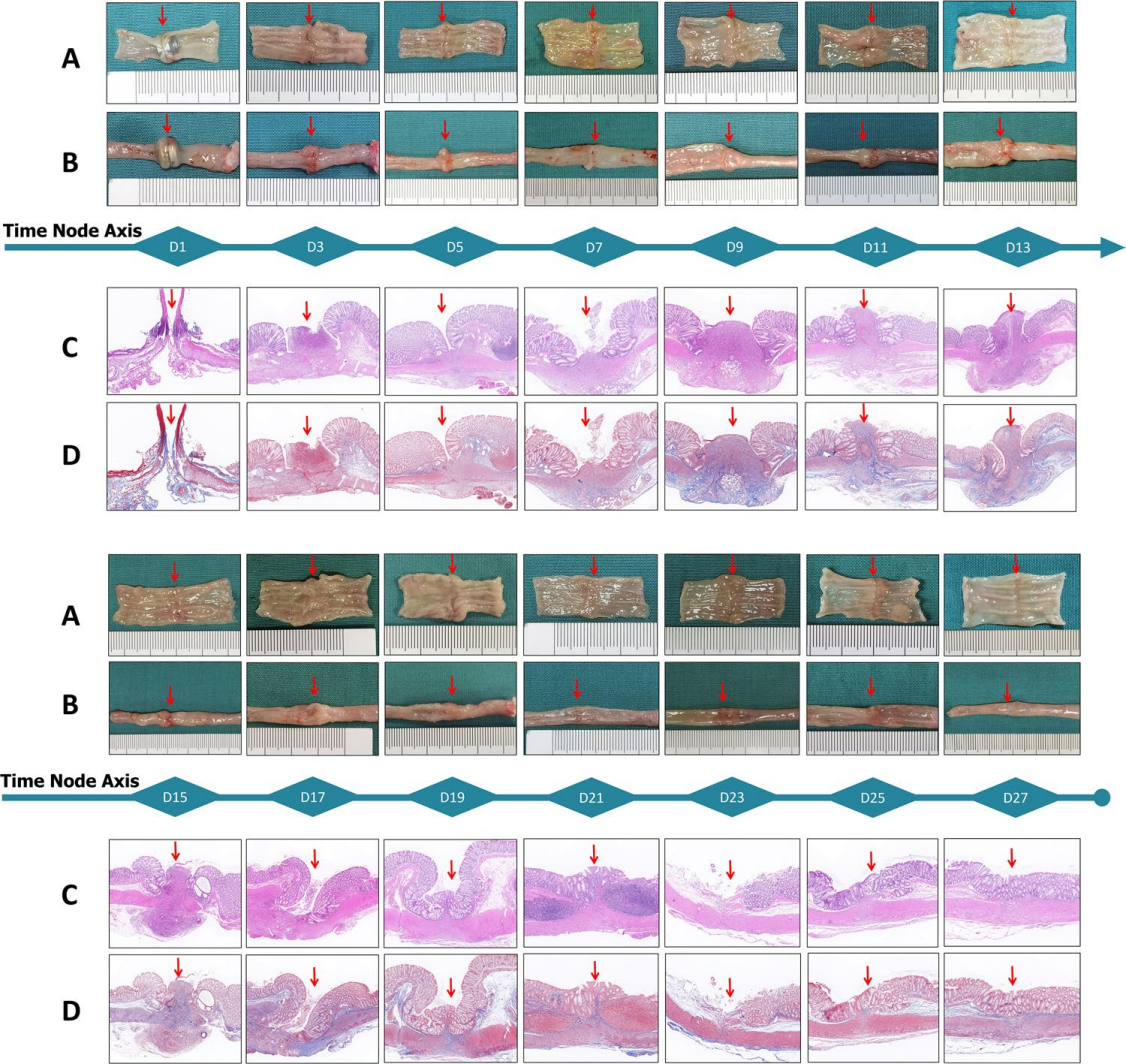
Gross examination showing the anastomotic sites (arrows) on the mucosal side (**A**) and the serosal side (**B**). Histological findings at the anastomotic sites after HE staining (4 ×) (**C**) and Masson’s staining (4 ×) (**D**).

Upon gross examination, the magnetic rings were firmly attached to each other at the anastomotic site in all rats in the D1 group and were difficult to detach. In the D3 group, the magnetic rings of 3 rats were located at the anastomotic site but the rings fell off after applying slight external force. The magnetic rings in the remaining 3 rats in the D3 group had separated from the anastomosis and were located in the rectum. In the D5 group, the magnetic rings were not present at the anastomotic position in any of the rats (Fig. 4**).**

Gross examination of the specimens showed that anastomosis was not established on POD1. The continuity of the intestinal lumen was maintained by the magnetic force between the magnetic rings. The serosal continuity was established on POD3, but the mucosal continuity at the anastomotic site was interrupted and termed "anastomotic sulcus". The anastomotic sulcus gradually became shallower by POD11 and disappeared by POD21 with the establishment of mucosal continuity.

Histopathological examination showed that the serosal and mucosal layers of the colon at the anastomotic site were interrupted on POD1 and POD3. In the specimens examined at POD3–POD11, the serosal layer at the anastomotic site was continuous but the continuity of the mucosal layer was not established. Starting from POD11, the continuity of the mucosal layer was gradually established and completed by POD21.

## Discussion

Since the first report of MCA in 1978 by Obora^14^, MCA has gained the attention of surgeons as a novel technique for anastomosis. There are many reports on the basic research and clinical applications of magnetic anastomosis^15–17^. However, the main concerns of MCA include the design of the MCA device, development of innovative surgical methods to perform MCA, and evaluation of anastomotic effects of MCA. Moreover, there are few studies on the mechanism of MCA, the magnetomechanical properties, and the histopathological changes at the anastomotic site. Previous studies have reported some problems with MCA such as narrow anastomoses and prolonged magnet expulsion time after surgery^18^. In order to overcome these problems, it is important to understand the pathological changes occurring at the anastomotic stoma with magnetic compression.

The skin wound healing process can be divided into three stages: inflammation, proliferation, and maturity. Clinical treatment differs based on the healing stage. Hence, knowing the various stages of MCA is important for clinical practice. In this study, the blast pressure of the digestive tract magnetic anastomosis specimen was measured and correlated with the macroscopic and microscopic changes occurring at the anastomotic site. The anastomotic bursting pressure was at a medium level before the magnetic ring was separated from the anastomosis. Once the magnetic ring was detached from the anastomosis, the blast pressure was reduced to a low level. With time, the bursting pressure gradually increased and plateaued at POD21. Gross and histological examinations of the anastomotic stoma showed development of an obvious circular depression called the anastomotic sulcus at the anastomotic site in the early stage after the separation of the magnetic ring from the anastomotic stoma. Microscopic examination showed that the serosal layer of the digestive tract healed, while the mucosal continuity at the site of anastomotic sulcus was not yet established, which is consistent with the trend of the anastomotic bursting pressure. Therefore, based on these observations, the digestive tract magnamosis can be divided into magnetic maintenance (serosal adhesion), fragile (serosal healing), strengthening (mucosal healing), and stable (modeling) stages. Such staging clarifies the dynamic changes occurring at the anastomotic site during the process of MCA.

In this study, rat colonic anastomosis was used as the research model for MCA. This model has the following advantages: rats are easy to raise and observe; rat colonic magnetic anastomosis is easy to perform; and the tissue specimens are easy to obtain. However, using a rat model for digestive tract MCA has the following disadvantages: rats have small intestinal tract lumens, thin intestinal walls, and short magnet discharge times compared to large animals and humans. Therefore, the staging proposed in this study cannot be extrapolated to larger animals and humans. Hence, future studies on digestive tract magnetic anastomosis in dogs and pigs would be useful to precisely determine the timing of changes occurring at the anastomotic site, which will be of great significance for guiding clinical practice. Furthermore, the role of micro-vessels and various cell subpopulations in the anastomotic tissues were not explored in this study. The study of the microstructural changes in the anastomotic tissues will help to better understand the process of magnamosis.

## Conclusions

In conclusion, we propose the use of Yan-Zhang’s staging system of digestive tract MCA based on burst pressure and pathological changes at the anastomotic site. To the best of our knowledge, this is the first published digestive tract MCA staging system. Future studies in larger animals are required to validate the findings of this study. While further optimization is required, our model provides significant insight about the dynamic process of digestive tract magnetic anastomosis.

## Data Availability

The data underlying this article will be shared on reasonable request to the corresponding author.
